# Chronic myeloid leukemia stem cells: targeting therapeutic implications

**DOI:** 10.1186/s13287-021-02659-1

**Published:** 2021-12-18

**Authors:** Hanieh Mojtahedi, Niloufar Yazdanpanah, Nima Rezaei

**Affiliations:** 1 Department of Immunology, School of Medicine, Shahid Beheshti University of Medical Sciences, Tehran, Iran; 2 Research Center for Immunodeficiencies, Children’s Medical Center Hospital, Tehran University of Medical Sciences, Dr. Qarib St, Keshavarz Blvd, 14194 Tehran, Iran; 3 Network of Immunity in Infection, Malignancy and Autoimmunity (NIIMA), Universal Scientific Education and Research Network (USERN), Tehran, Iran; 4 School of Medicine, Tehran University of Medical Sciences, Tehran, Iran; 5 Department of Immunology, School of Medicine, Tehran University of Medical Sciences, Tehran, Iran

**Keywords:** Chronic myeloid leukemia (CML), Leukemia stem cells (LSCs), CML LSCs, BCR-ABL, Tyrosine kinase inhibitors (TKIs)

## Abstract

Chronic myeloid leukemia (CML) is a clonal myeloproliferative neoplasm driven by BCR-ABL1 oncoprotein, which plays a pivotal role in CML pathology, diagnosis, and treatment as confirmed by the success of tyrosine kinase inhibitor (TKI) therapy. Despite advances in the development of more potent tyrosine kinase inhibitors, some mechanisms particularly in terms of CML leukemic stem cell (CML LSC) lead to intrinsic or acquired therapy resistance, relapse, and disease progression. In fact, the maintenance CML LSCs in patients who are resistance to TKI therapy indicates the role of CML LSCs in resistance to therapy through survival mechanisms that are not completely dependent on BCR-ABL activity. Targeting therapeutic approaches aim to eradicate CML LSCs through characterization and targeting genetic alteration and molecular pathways involving in CML LSC survival in a favorable leukemic microenvironment and resistance to apoptosis, with the hope of providing a functional cure. In other words, it is possible to develop the combination therapy of TKs with drugs targeting genes or molecules more specifically, which is required for survival mechanisms of CML LSCs, while sparing normal HSCs for clinical benefits along with TKIs.

## Introduction

Chronic myeloid leukemia (CML) is a clonal myeloproliferative neoplasm featured with uncontrolled proliferation of myeloid cells at every stage of differentiation [1]. CML is the first discovery of a link between cancer and chromosomal abnormality, which also called the Philadelphia (Ph) chromosome, due to the reciprocal translocation that happens between the Abelson murine leukemia virus (*ABL*) gene on chromosomes 9 and the breakpoint cluster region (*BCR*) on chromosomes 22 (t9;22) (q34;q11) [2]. The presence of fusion oncogene BCR-ABL1 and generating a 210 KD chimeric oncoprotein (P210) with the constitutive activity of tyrosine kinase leads to clonal expansion of hematopoietic stem cell (HSC) and the pathogenesis of CML [3]. BCR-ABL1 contributes to CML leukemia stem cells (LSCs), identified by survival promotion, the capacity of self-renewal, and differentiation to aberrant hematopoietic subsets and resistance to apoptosis through the activation of various signaling molecules and pathways in downstream of the BCR-ABL protein, to regulate leukemogenesis [4, 5]. The LSCs can be also a persistent problem of CML patients since regardless of BCR-ABL kinase activation, they alter the relationship with bone marrow niche and leave the quiescence state and reach to proliferation state via intracellular signaling changes and suppressive cytokines secretion that can disrupt metabolic processes, prepare an anti-apoptotic microenvironment, and dysregulate immunological activities [6].

Allogeneic transplantation (bone marrow (BM)/HSC transplantation) is considered as the curative treatment of CML, although finding a suitable donor is the problematic factor for the treatment. In recent years, the discovery of imatinib mesylate (IM), as the first designed BCR-ABL1 tyrosine kinase inhibitor (TKI) and additional second- (dasatinib, nilotinib (NIL), and bosutinib) and third-generation (ponatinib) TKIs have dramatically enhanced the chance of survival in CML patients [7]. However, the major barrier against treatment of CML via the family of TKIs is primary and acquired resistance resulted from the accumulation of LSCs with a high heterogeneity in the blood and BM [8]. In other words, CML therapies including cytotoxic chemotherapy and TKIs cannot kill the population of quiescent LSCs, which are in charge of the subsequent relapse and CML stage progression. CML is a triphasic stage disease that begins with a chronic phase (CP), which is characterized by a significant increase in myeloid precursors and lasts about 3–5 years; however, without therapeutic intervention or in cases of refractory disease after 3-18 months, the disease progresses spontaneously toward accelerated phase (AP), and eventually to highly aggressive blast crisis phase (BCP), with a rapid expansion of primary bone marrow cells that spread to the circulatory system [9, 10].

In brief, CML stem cells can be either expressive or independent of BCR-ABL1 and represent TKI-resistant [11]. Therefore, the discovery of uncertain CML LSCs resistance mechanisms can be a great target to facilitate the eradication of these cells. This review underlines the current known characteristics of CML LSCs besides possible mechanisms and pathways that lead to survival and resistance to targeted multimodal therapies that might help developing new drugs and treatment options, since current therapeutic strategies often represent limitations and failures.

## Origins and evolution of CML LSCs

In 1960, an abnormal “minute chromosome” which later became known as the “Philadelphia (Ph) chromosome” was identified in patients with CML by Peter Nowell and David Hungerford, representing the first discovery of a link between chromosomes and cancer [12]. Thirteen years later, Janet Rowley observed that the Ph chromosome was the result of the reciprocal translocation between chromosomes 9 and 22 which usually takes place in HSCs as the top of the hematopoietic hierarchy [13]. Over ten years later, it became evident that the human homolog of the Abelson murine leukemia viral oncogene (*ABL1)* in the region of chromosome 9 translocates to breakpoint cluster region (BCR) on chromosome 22 [14, 15]. In the early 1990s, the first convincing evidence of CML stem cells came from early observations in which transfusion of unirradiated white blood cells obtained by leukapheresis from CML patients with high granulocyte counts into severely neutropenic recipients, resulting in the transient repopulated Ph^+^/*BCR*-*ABL*^+^ cells [16]. In the following years, it is believed that the cell of origin in CML is characterized through specific genomic alteration of the CD34^+^CD38^−^ subset of HSCs leading to Ph chromosome and the expression of fusion tyrosine kinase BCR-ABL1 and leukemogenesis [9]. However, the BCR-ABL1 translocation can be formed to three types of chimeric proteins with different mass and biological features as a result of breakage in one of the three regions of the *BCR* gene, named major (M-bcr), minor (m-bcr), and micro-bcr (µ-bcr) [17]. Fusion of *BCR* exon 13 or 14 (e13/e14) with *ABL1* exon 2 (a2) forms a fusion gene referred to as e13a2 (b2a2) or e14a2 (b3a2), leading to the P210^BCR-ABL1^ protein (M-bcr) seen in the majority of CML patients [17, 18]. In rare cases of CML and two-thirds of Ph-positive acute lymphoblastic leukemia (Ph^+^ ALL), the breakpoint occurs in the first intron on the *BCR* gene (m-BCR), creating the P190^BCR-ABL1^ from the e1a2 transcript. Approximately one-third of Ph^+^ ALL patients will express one of the longer BCR-ABL1 isoforms [17–19]. In 2009, both Mullighan *et al.*, and Den Boer *et al.* described a new subtype of ALL, named Philadelphia chromosome-like (Ph-like) or BCR-ABL1-like ALL, respectively [20, 21]. In 2016, the World Health Organization (WHO) classifies BCR-ABL1-like ALL as a provisional entity of hematopoietic neoplasm with a lack of the BCR-ABL1 translocation (about 90%) but having a highly similar profile of gene expression to that seen in ALL with BCR-ABL1 (Ph^+^ ALL ) and also a high frequency of deletions of IKAROS family zinc finger 1 (IKZF1) [22]. Overall, BCR-ABL1 fusion is present in essentially all cases of CML and in about 3-5% of pediatric ALL and 25% of adult ALL (mostly B-ALL) [22], while BCR-ABL1-like ALL subset of patients goes for about 20% of B-ALL cases overall. Moreover, about 1% of all patients with acute myeloid leukemia (AML) have Philadelphia translocation, which is cytogenetically indistinguishable from Ph^+^ CML; meanwhile, the breakpoint on chromosome 22 in Ph^+^ AML is distinct from CML [23, 24].

In 1986, BCR-ABL1 molecular aberration defines as the start-up of a chronic phase (CP), which in the absence of effective therapy, leads toward acute phase by further genetic instability and acquisition of mutations as a result of the P210^BCR-ABL1^ activity [25, 26]. All CML patients in blast crisis phase co-express both co-express P210^BCR-ABL1^ and P190 BCR-ABL1 transcripts [27]. Mutations in ABL1, IKZF1, RUNX1, ASXL1, and TP53, including an overrepresentation of ABL1 and IKZF1 mutations in lymphoid blast crisis, are among the additional genetic aberrations that occur during progression to accelerated phase and aggressive blast crisis [28]. However, the detection of low levels of BCR-ABL1 transcripts in healthy individuals [29] implies that a translocation has happened in a cell that does not possess the ability to initiate leukemia. In other words, BCR-ABL is required but not sufficient for the production of CML LSCs, which may necessitate further BCR-ABL copy number multiplication, subsequent mutations, and genomic instability [9]. Over the total genetic alterations of HSCs into CML LSCs, activation of various signaling pathways leads to CML progression as an LSC-derived disease, along with dysfunction of underlying cellular processes.

## Properties of CML LSCs

A main challenge in developing strategies to provide novel ways for therapeutic targeting of CML LSCs is how to isolate and characterize these cells to increase therapeutic effects and reduce toxicities. One of the ways to identify is extensive analyses of unique cell surface antigens on CML LSCs, which must be specific or have a distinct expression pattern, density, or dispersion. Besides, similar to normal HSCs, CML LSCs can self-renew, differentiate, or enter dormancy. Moreover, knowing signaling pathways and mechanisms involved in the differentiation and survival of CML LSCs is another step to develop different therapeutic strategies to eliminate them [11].

### Cell surface markers

CML LSCs share many similarities with HSCs including cell surface phenotype such as Lin^−^CD34^+^CD38^−^. Many markers are reportedly expressed specifically on CML LSCs, including CD25, CD26, and interleukin-1 receptor accessory protein (IL-1RAP), which can be used as biomarkers for diagnostic, prognostic, or therapeutic purposes [30–32]. The increased expression of CD25 is related to the reduction in the proliferation capacity of CML LSCs and CML disease progression. Therefore, CD25 (IL2RA) is suggested as a marker of CML LSCs and a negative regulator of growth [33]. Furthermore, the IL-2-CD25 axis plays a critical role in the preservation of CML LSCs by a massive expansion of the LSCs during TKI resistance, which makes CD25 a novel marker and drug-sensitive suppressor in CML therapy [34, 35]. IL-1RAP, as an interleukin 1 receptor-1 (IL1R1) coreceptor, leads to increased proliferation of CML LSCs by binding of IL-1A or IL-1B and activation of the NF-κB, JNK, and P38-MAPK signaling pathways [33]. CD26 (dipeptidyl peptidase-4 (DPP4)) is involved in releasing CML LSCs into the bloodstream from the BM through cleaving stromal-derived factor (SDF-1**:** CXCL12)-CXCR4 (CD184) axis, which can lead to the disruption of interactions within the stem cell niche and spread of the disease regardless of specific niche regulations. Furthermore, as TKI resistance improved, the number of CD26^+^ CML LSCs increased [36]. Additionally, only CD26 appears to offer clinical application, although, in the preclinical era, other biomarkers of CML LSCs have been discovered [37]. CD33 (Siglec-3) expressed at greater levels in CML LSCs [38]. CD36 (scavenger receptor-B2 (SR-B2)) is a marker of immature CML cells that its upregulated expression on CD34^+^CD38^low^ CML cells in chronic phase leads to less sensitivity to IM treatment [39]. In addition, in blast crisis CML, CD36 has indicated the mediation of fatty acid uptake with distinct metabolic properties [40]. There are other additional antigens, including CD44, CD47 (IAP), CD529 (Campath-1), CD56 (NCAM), CD90 (Thy-1), CD93, CD114 (granulocyte colony-stimulating factor (G-CSF) receptor), CD123 (IL-3RA), CD135 (FMS-like tyrosine kinase 3 (FLT3)), and CD295 (LEPR) that are also present on CML LSCs [34, 41–43]. CD44 is important for the engraftment of CML cells by its interaction with different bone marrow microenvironment (BMM)-associated proteins such as hyaluronan, osteopontin, or E-selectin [44]. The expression of CD93 is constant and selective in the subset of CML LSCs, which indicated self-renewal and proliferation capacity of this primitive cell subpopulation by the expression of selected genes such as *ID2*, *MYB*, *PAK2*, *MEIS1*, *REL*, *CK1, CDK4*, and *CCND2*. Furthermore, CD93, as a major integrin α5β1 mediator, along with CD44 can bind to fibronectin [45, 46] and might have a role in LSC adherence within the BM niche [37, 44]. Although CD93 is consistently and selectively expressed on CML LSCs, it can also be found on other cell types such as platelets and endothelial cells; therefore, CD93 is unlikely to serve as a therapeutic target, while it can be used as a prognostic biomarker to differentiate CML patients at high risk for molecular relapse after treatment discontinuation [47]. The possible profile of LSCs in CML could be Lin^−^CD34^+^CD38^−^CD45RA^−/Low^, CD117 (KIT)^−^, CD26^+^, and CD90^+^ that are identified as potential therapeutic targets at a single-cell level with a higher proliferative and colony-forming potential than normal HSCs [37, 48]. It has been reported that stem cell factor receptor (SCFR), also known as c-Kit receptor or CD117, was expressed on CD34^+^CD38^low^ CML cells, although healthy cells have represented a greater level of SCFR expression [49]**.** However, the precise immunophenotype of these primitive CML cells is not completely determined, while the discovery of novel cell surface antigens on primitive CML cells might lead to novel treatment strategies.

### Self-renewal and differentiation

LSCs have a self-renewal capacity to generate large numbers of leukemia progenitor cells (CD34^+^CD38^+^) through the activation of several signaling pathways including WNT/β-catenin, Hedgehog (Hh), Notch, transforming growth factor-β (TGF-β)/Forkhead box O (FOXO), and Musashi 2 (Msi2)-Numb signaling [50]. The P210^BCR−ABL1^ also regulates miRNA stability and induces an increase in self-renewal of CML LSCs by mediating the activation of Janus kinase (JAK)/STAT signaling pathway, increasing adenosine deaminases acting on double-strand RNA1 (ADAR1) enzyme levels, which hampers biogenesis of the miR precursor (miR-let7) and increased LIN28B pluripotency gene expression [51, 52]. Moreover, the expression of the krüppel-like factor 4 (KLF4) is essential for the regulation of LSC self-renewal and maintenance of CML. In this way, in the absence of KLF4 in CML LSCs, dual specificity tyrosine phosphorylation regulated kinase 2 (DYRK2) was remarkably upregulated, which in turn mediated the activation of P53 and c-MYC, and facilitated proteasomal degradation through phosphorylation, leading to apoptosis and decrease in the self-renewal in CML LSCs [53]. BMI1, a polycomb protein belonging to the polycomb repressive complex 1 (PRC1) family, works with BCR-ABL and is also involved in proliferation and self-renewal of LSCs [54]. MicroRNAs may be effective in self-renewal and maintenance of CML LSC, in particular miR-126, which regulates the dormancy of normal stem cells including CML LSCs [55]**.** CML stem cells could be able to maintain the energy output required for self-renewal capability by utilizing anaplerosis, which is a series of metabolic reactions in which critical TCA cycle intermediates, especially oxaloacetic acid, are produced [56]. BCR-ABL1 oncoprotein stimulates CML LSC self-renewal by increasing the expression of protein phosphatase 2A (PP2A) inhibitors, such as protein SET (SET), cancerous inhibitor of PP2A (CIP2A), and PP2A-Aα, resulting in inactivation of PP2A and MYC overexpression, LSC survival, and the establishment of a positive feedback loop for CML development to BP [57]. Besides, the high expression and activation of viral integration site 1 (EVI1) in CD34^+^CD38^–^ stem cells leads to increase self-renewal capacity, leukemia development and the disease progression to BP [58]. LIGHT/lymphotoxin-β receptor (LTβR) signaling regulates quiescence and self-renewal of LSCs and HSCs by reducing cell proliferation and favoring symmetric cell division over asymmetric division. Since asymmetric division leads to differentiation and exhaustion of stem cells, targeting the LIGHT/LTβR pathway may offer a novel strategy to induce differentiation and eliminate LSCs [59].

CML LSCs in chronic phase can simultaneously differentiate into a variety of malignant leukocytes that are morphologically mature and molecularly expressing BCR-ABL. As a result, the leukocyte population in CP are heterogeneous, with a limited number of LSCs and the bulk of leukemia non-stem cells [60]. Alternatively, leukemia non-stem cells including basophils and megakaryocytes (the platelet’s precursor) can play a role in CML pathogenesis and development [61]. Basophil-like CML cell-derived mediators such as hepatocyte growth factor (HGF) [62] and tryptase [63] might possibly trigger CML progression by altering the bone marrow niche, leading to increased fibrosis and a supportive growth environment for LSCs, as well as production of CCL3, which can facilitate CML LSCs proliferation [61]. BCR-ABL-expressing megakaryocytes in CML can induce senescence along with expression of TGF-β1 in a P16- and P21-dependent manner, supporting the maintenance of CML LSCs [64]. Furthermore, uncontrolled expression of c-MYC/miR-150 in LSC and Ph^+^ cells downregulates BCL-2 inhibitor (miR-153-3p), resulting in impairment of myeloid differentiation and increase TKI resistance [65]. It has been proposed that the enhancement of self-renewal and compromising differentiation can affect disease progression that appears to result from aberrant activation of self-renewal signaling pathways in a progenitor cell population or failure to shut down these pathways during differentiation processes. Therefore, they might be a promising therapeutic target for CML LSCs.

## Survival and functional resistance of CML LSCs to therapies

Several factors are suggested to be important in the maintenance of CML LSCs through either the direct activation by kinase activity of BCR-ABL or independent of BCR-ABL kinase activity [6]. CML LSCs could avoid immune surveillance via a number of molecular pathways [66]**.** The other required factors in survival of CML LSCs are bone marrow resident cells and the support of LSCs in localization to the bone marrow niche [67]. It is thought that intrinsic regulatory systems and extrinsic microenvironmental signals are responsible for controlling quiescence of LSCs. In addition, the connection with the bone marrow niche and signaling molecules associated with self-renewal and survival are two key components in the long-term persistence of quiescent LSCs in CML, resulting in resistance to targeted therapy and increased risk of disease recurrence following therapy withdrawal [68, 69].

### Bone marrow microenvironment (BMM)

Numerous types of cells in the bone marrow niche, including osteoblasts, endothelial cells, mesenchymal stromal/stem cells (MSCs), megakaryocytes, and neural cells, interact with normal HSCs via various mediators and signaling pathways [50]. BCR-ABL1^T315I^ mutation leads to the maintenance of quiescent stem cells in the presence of TKIs through alternations in niche localization and increased level of integrin β3 and integrin-linked kinase (ILK) [70]. LSCs engraft in a different way than common HSCs, using distinct niche signals. There are controversial results regarding homing of CML LSCs, which displays impaired homing and persistence in the BM by inhibiting CXCL12 expression [71]. On the contrary, the elevated level of CXCR4 protein drives CML LSCs homing into the BMM leading to quiescence and TKI resistance. Hence, the decreased level of CXCR4 as a result of the inhibition of BCR-ABL1 tyrosine kinase activity can cause distribution of CML LSCs into the peripheral blood from the BMM [33]. Malfunction of β1-integrin (VLA4 or VLA5) causes redistribution of CML stem cells into the peripheral blood and different organs, which can potentially lead to uncontrolled extramedullary myeloproliferation and local LSC pools [72]. Multiple adhesion molecules expressed by LSCs, such as cadherins, CD44, and galectin-3, can also aid in the localization of LSCs to bone marrow niche to survive [61].

Furthermore, bone marrow resident cells support CML LSCs persistence by releasing various soluble substances such as JAGGED1 (a Notch ligand), TGF-β, bone morphogenic proteins (BMPs), chemokines, CXCL12, IL-1, and exosomes containing miR-126 [61]. Response of CML LSCs to the BMP/TGFβ superfamily is dysregulated to make the BMM more suitable for their survival by overexpression of the BMP type I receptors (BMBR1B) [73]. Furthermore, stimulation of the Notch signaling pathway in the BMM by the overexpression of Jagged2 reduces CML LSC cycling, indicating that Notch signaling fosters an antileukemic milieu, although the overexpression of Notch targets HES1 in CML patients in blast crisis, indicating that it may play a role in the development of CML from chronic phase to BP. As a result, the effects of Notch in CML LSCs need to be further investigated [74]. Altogether, the increased expression of BMPR1B, BMP2/4, and the embryonic transcription factor TWIST1, as well as the co-activation of SMAD1/5/8 and JAK2/STAT3 signaling are involved in LSC survival, treatment resistance and disease progression [75–77].

The CML-derived microvesicles (MVs) represent communication vehicles with the microenvironment which can shuttle miRNAs, amphiregulin, as well as BCR-ABL mRNAs to close stromal cells and cause reprogramming of niche cell functions [78]. CML-derived exosomes transfer miR-92a and activate SRC signaling, which induces phosphorylation of AKT and ERK1*/*2 and oncogenic signaling by BCR-ABL. However, this activation process can be reduced by TKI treatment [79, 80]. In addition, the secretion of exosomes containing amphiregulin along with CML LSCs can promote CML LSCs adhesion and their survival by activation the epidermal growth factor (EGFR) pathway in MSCs and increase secretion of IL-8, which stimulates human vascular endothelial cells to enhance the expression of intercellular adhesion molecule-1 (ICAM-1) and vascular cell adhesion molecule-1 (VCAM-1) [81, 82]. MSCs also play a role in maintaining CML stem cells in the BMM and TKI resistance through the upregulation of promyelocytic leukemia (PML), a tumor suppressor protein, and the cell quiescence regulator, to repair double-strand DNA breaks in MSCs that are used to upregulate inflammatory cytokines such as IL-6, IL-8 (CXCR2L) and CXCL1/CXCR2 as well as activation of various signaling pathways [83]. Therefore, targeting CXCR2 signaling might be a novel targeted therapy in TKI-resistant CML patients through the suppression of AKT/mTOR and c-MYC [84]. Moreover, MSCs can cause an increase in proliferation, self-renewal, and anti-apoptotic capability of CML LSCs and TKI resistance by the secretion of exogenous WNT and activation of WNT/β-catenin signaling during progression to the advanced phase of the disease [85–87]. The production of fibroblast growth factor 2 (FGF2) by BM-derived MSCs and the decreased level of reactive oxygen species (ROS) in CML LSCs also increases TKI resistance [88, 89].

Other way to enhance survival of CML progenitors is the reduced oxygen in the microenvironment formed a gradient of oxygen from the blood vessel to hypoxic areas [90]. In hypoxic BMM, there are increased levels of hypoxia-inducible factors (HIFs), being essential for regulation of proliferation, quiescence, TKI resistance, and survival of CML LSCs, as well as increased capacity of HSCs to transform into LSCs, mediated by increased glycolytic flux via glucose transporter 1 (GLUT1) and tumor M2-pyruvate kinase (PKM2), overexpression of P21, suppression of P53, increased transcription of antioxidant factors (FOXO and NRF2), and uptake of c-MYC [91–93]. In order to adjust to low oxygen concentrations in the BMM to facilitate dormancy and self-renewal of CML LSC, the STAT5/HIF-2α/CITED pathway is activated. However, peroxisome proliferator-activated γ (PPARγ), a negative regulator of this pathway, inhibits LSCs adhesion to the extracellular matrix and drives apoptosis [94, 95].

### Autophagy

Autophagy is a vital pathway for survival and drug resistance of BCR-ABL1 expressing cells, resulting in reduced P53-mediated stress response and pro-apoptotic signals to avoid cell death, although the constitutive activation of BCR-ABL kinase can downregulate autophagy by activation of the phosphoinositide 3-kinase (PI3K)/AKT/FOXO4 pathway and the upregulation of the mTOR pathway [96, 97]. The increased expression of autophagy genes such as autophagy-related 4B cysteine peptidase (*ATG4B*) in CD34^+^ CML cells indicates that *ATG4B* may be a potential target for CML LSCs that its knockdown impairs proliferation and survival of CML stem cells [98]. Autophagy can also be linked to metabolism; in this way that knockdown of *ATG7* caused a reduction in glycolysis, an elevation in oxidative phosphorylation, and an accumulation of mitochondrial ROS in CML cells [76].

### Energy metabolism

In order to induce the survival and maintenance of stemness in LSCs, the metabolic alteration results in increased glucose flux, glycolysis, pyruvate transferring, anaplerosis, oxidative phosphorylation, and ROS accumulation [99]. BCR-ABL1 can stimulate aerobic glycolysis (the Warburg effect) in CML cells through activation of the PI3K/AKT pathway [100]. CML LSCs also represent a drastic increase in mitochondrial oxidative phosphorylation, which was controlled by activation of oxidative metabolism genes [56]. Interestingly, STAT3 has been connected to mitochondrial respiration; thus, it may assume that STAT3 involved in abnormal energy metabolism in CML LSCs and TKI-resistant. STAT3-mediated metabolic regulation might link to BMM-mediated quiescence and survival of LSCs [101, 102].

Fatty acid metabolism is also important for CML LSC survival [103]. CML LSCs show greater lipolysis and fatty acid oxidation capacity in comparison with differentiated CML cells [40, 56]. Fatty acid metabolism may be an additional factor in increasing the dependence of myeloid LSCs on oxidative phosphorylation. However, redundancy in fatty acid metabolism and signaling pathways, as well as a multiplicity of various fatty acid-binding proteins, may complicate targeting this process in LSCs [102]. Arachidonate lipoxygenase (ALOX5)-5 and ALOX15 are also involved in LSC self-renewal and fatty acid metabolism by changing arachidonic acid to leukotrienes such as leukotriene B4 (LTB4), which increases the expression of the leukotriene B4 receptor 2 (BLT2) in CML stem cells [104, 105]. Moreover, lysophospholipid metabolism plays a part in the maintenance of CML stem cells and TKI resistance by regulation of lysophospholipase D activity of GDPD3 in the BCR-ABL1-independent manner signaling pathway, and FOXO3A/β-catenin interaction [106].

In the hypoxia condition, metabolic reprogramming of CML LSCs is a pathway of BCR-ABL1-independent TKI resistance. The elevated amount of intracellular dipeptide amino acids in CML LSCs can cause CML LSC maintenance by the stimulation of the P38/MAPK pathway and phosphorylation of SMAD3/FOXO3A [107]. Besides, CML LSCs under hypoxic conditions induces upregulation of genes involved in carbohydrate metabolism to support energy production. Various enzymes associated with glucose metabolism are able to mediate CML cell survival despite TKI-mediated BCR-ABL1 inhibition, including the enzyme dihydrolipoamide S-acetyltransferase (DLAT), involved in the pyruvate dehydrogenase (PDH) complex [90]. The activation and upregulation of BCAA transaminase 1 (BCAT1), a cytosolic aminotransferase for branched-chain amino acids (BCAA), by Musashi2 is functionally needed for CML progression and metabolic reprogramming. The increased BCAA metabolism triggers activation of mTORC1 and increase BC-CML-initiating cells survival; hence, blocking BCAA metabolism and enzymatic activity of BCAT1 inhibits proliferation and causes cellular differentiation in blast crisis CML cells [103]. However, further research is needed to understand how CML LSCs become dependent on oxidative metabolism for survival.

### Signaling pathways and potential molecules

**Fig. 1:**
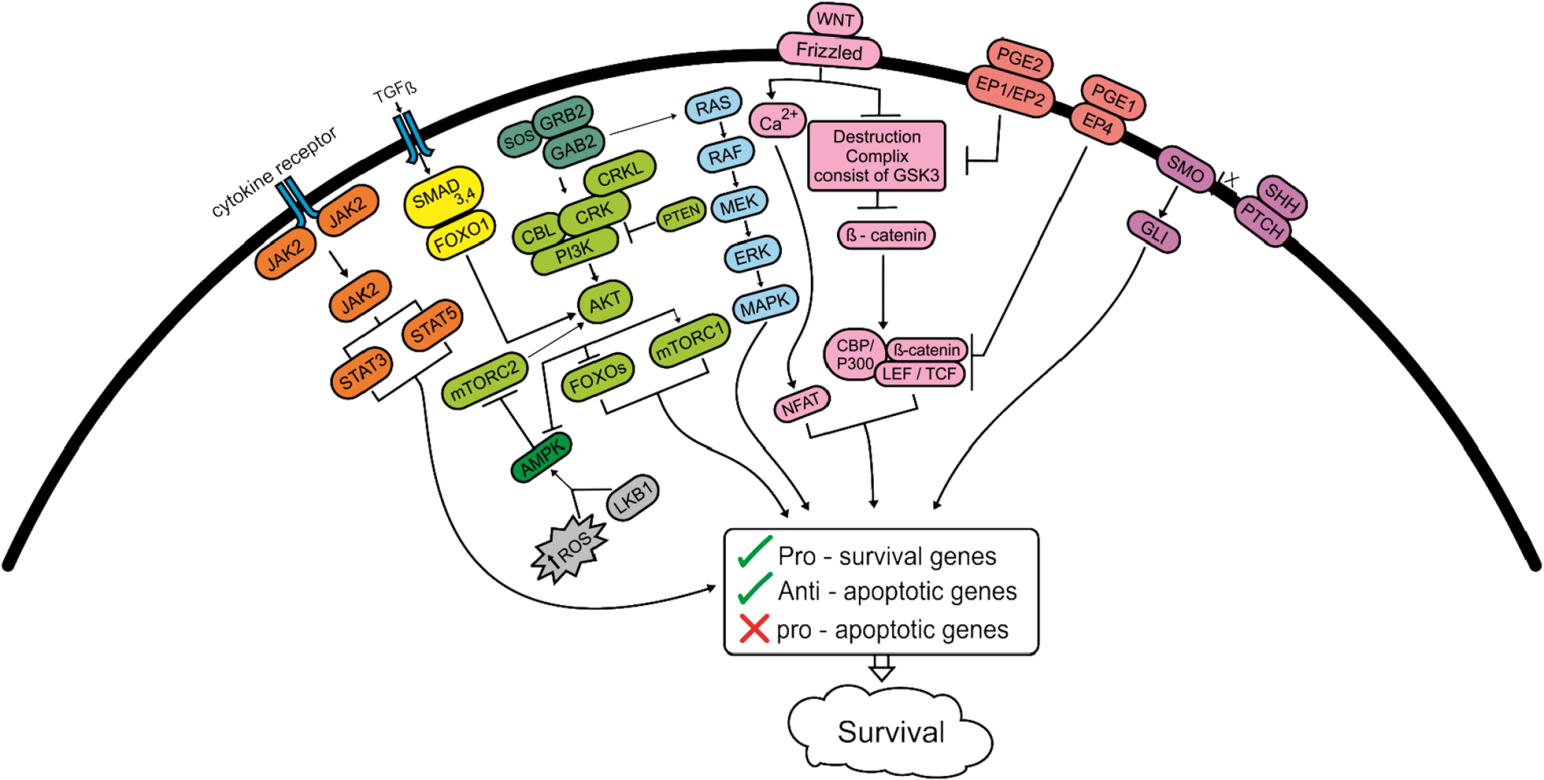
Role of signaling pathways in survival of CML LSC

The generation of *BCR-ABL* fusion gene in CML LSCs may have a role in a variety of biological processes, notably cell proliferation, differentiation, and apoptosis by encoding a constitutively activating tyrosine kinase as important mediator of signaling cascades such as RAS/mitogen-activated protein kinase (MAPK), PI3K/Akt/FOXO axis, WNT signaling, and JAK2/STAT3,5 signaling, which are implicated in the maintenance and survival of CML LSCs (Fig. 1) [61, 108]. As a result of the signaling pathways upregulation, the level of ROS and genomic instability increased that have a potential to create other mutations and chromosomal abnormalities, following the advancement of CML from the CP to the BP [109, 110].

The high-affinity attachment of the growth factor receptor-bound protein 2 (GRB2) and the scaffolding protein Gab2 following the phosphorylation of tyrosine residue domain SH2 of *ABL* activates the Ras pathway [111]. Activation of RAF/mitogen-activated protein kinase (MAPK)/extracellular signal regulated kinase (ERK) signaling pathway in a RAS-dependent manner plays an important part in BCR-ABL-independent TKI resistance through an increase in the expression of protein kinase C (PKC) and protein kinase C eta (PRKCH), leading to additional proliferation along with the inhibition of CML stem cells apoptosis by the activation of transcription factors including c-JUN, c-MYC, and c-FOS, as well as the expression of genes involved in proliferation processes [17, 112]. Although for sustaining the proliferative properties of BCR-ABL^+^ cells, an active PI3K is required [17]. Moreover, the oncoprotein BCR-ABL can activate PI3K and AKT-mediated phosphorylation and cytosolic retention of FOXO transcription factors in different ways such as the association of the SRC homology 2 domain-containing (SHC) protein binding to the P85 regulatory subunit [113], besides binding to GRB2 [114] and the formation of complex c-CBL and adapter molecules (CRK/CRKL). The upregulation of FOXO target genes (e.g., the pro-survival factor BCL6, ATM, and cyclin-dependent kinase 1 (CDK1)) results in blocking the interaction of pro-apoptotic BAD proteins with anti-apoptotic proteins such as BCL-2 and B-cell lymphoma-extralarge (BCL-XL) and no release of pro-apoptotic factors besides the activation of caspase cascade. Therefore, BCR-ABL can directly activate and increase the expression of anti-apoptotic BCL-2 family members such as MCL-1 and BCL-XL, without the participation of JAK/STAT5 [96, 115, 116]. The pathway's third link is mTOR, which involves mTORC1, which is upregulated by AKT activation, and mTORC2, which mediates AKT activation [117]. In accordance with cross talk signaling, low level secretion of TGF-β by CML LSCs can support the survival of CML LSCs and leukemia progression through AKT activation, leading to FOXO3A cytosolic localization, mitochondrial dysfunction, and abnormal ROS production [118, 119]. Besides, the dysregulated PI3K/AKT/mTOR signaling in LSCs increases ROS production, which promotes the survival and drug resistance by the loss of negative the regulation of liver kinase B1 (LKB1) that controls the activity of AMP-activated kinase (AMPK) [117]. The activation of AMPK can induce caspase-3-mediated LSC apoptosis by upregulating P38 expression and c-JUN N-terminal kinase (JNK)-mediated phosphorylation of H2AX, in addition to induce downregulation of BCL-2 [120]. Overexpression of PTEN as a negative regulator of PI3K/AKT and downregulation of β-catenin expression by inhibiting AlOX15 expression can increase susceptibility of CML LSCs to TKI therapy and delayed disease progression [105].

The other signaling pathway in CML cells, which is mediated by binding of JAK2 to the SH2 domain of BCR-ABL1, leads to phosphorylation and activation of STAT3/5 [121]. The activated JAK/STAT signaling drives increased expression and activity of ADAR-1, which contributes to the malignant reprogramming of myeloid progenitors into LSC and CML development, as well as enhanced MDM2 expression and inhibition of the P53 tumor suppressor by creating misspliced isoforms of glycogen synthase kinase 3 beta (GSK3) [122]. The STAT3 activation confers survival and acquired TKI resistance of CML LSCs in BMM through BCR-ABL1-independent mechanisms, suggesting a different role for STAT3 in drug resistance of CML [123]. It has been observed that in both BCR-ABL-dependent STAT5-mediated pathway and a BCR-ABL-independent STAT4-mediated pathway, the expression of a pro-survival gene known as *PIM2* is increased in CML LSCs leading to inhibition of the pro-apoptotic protein BAD [124]. Moreover, signal-transducing adapter protein-1 (STAP-1) is important for the preservation of CML LSCs because it suppresses apoptosis by regulating and reducing STAT5 phosphorylation, either directly or indirectly, via the PPAR signaling pathway [125]. The constitutive activation of STAT5 due to the presence of the BCR-ABL oncoprotein mediates the expression of anti-apoptotic proteins and maintenance of CML LSCs [126].

The WNT signaling pathway is composed to the canonical or “β-catenin dependent” pathway, and the non-canonical "WNT/calcium^2+^/nuclear factor of activated T-cells (NFAT)" pathway or "β-catenin-independent" pathway. BCR-ABL1 can mediate TKI resistance by the induction of constitutive release of WNT ligands, overexpression of frizzled-4 (FZD4) receptors for stabilization of β-catenin in the nucleus and its binding to DNA by interactions with other transcription factors (such as TCF/LEF family and FOXOs), leading to constitutive activation of target genes, such as survival genes (c-MYC and cyclinD1) [127–129]. In fact, the activation of WNT/β-catenin mediates the nuclear translocation of NFκB-P65, and its interaction with FOXM1/β-catenin that is essential for the survival of CML LSCs [130]. β-catenin physically interact with CREB-binding protein (CBP)/P300, identified as transcriptional coactivators in cell differentiation, indicating that CBP inhibition may be a new strategy for CML therapy [131]. Moreover, the increased accumulation of β-catenin nuclear accumulation by upregulation of prostaglandin E2 (PGE2) and their binding to E-prostanoid 1/2 (EP1/EP2) receptor leads to the improvement of the β-catenin/WNT signaling, conferring to LSC stemness, resistance to TKI and CML development [33, 132].

Additionally, the activation and upregulation of the smoothened (SMO) transmembrane protein in hedgehog (Hh) pathway can play a potential role in the maintenance of stem cells in myeloid leukemia. In fact, the Hh pathway in CML LSCs is triggered by attaching a ligand, sonic hedgehog (SHH), to the PTCH receptor, which removes PTCH-mediated suppression of SMO, and a conformational change of SMO, which stimulates the glioma-linked oncogene (GLI1 and GLI2) transcription factor, leading to reduced apoptosis [133, 134]. The persistence of Hh signaling unlike TKI therapy implies that this pathway is BCR-ABL1-independent [133, 134], and CML LSCs can use this pathways along with ALOX5 for their survival and TKI resistance [104]. Msi2-Numb signaling as a molecular pathway is correlated with HH and Notch and is involved in regulating the self-renewal properties of LSCs [135].

### Epigenetic reprogramming

In addition to transcription factors, epigenetic regulation is other critical element for stem cell maintenance by modulating chromatin accessibility via DNA methylation and histone acetylation at gene expression, responsible for signaling pathways of survival, proliferation, and cell differentiation [136]. The dysregulation of leukemia-specific cellular pathways, such as an increase in the levels of cytidine deaminase and ROS lead to preleukemic lesions in epigenetic regulators (e.g., (cytosine-5)-methyltransferase 3A (DNMT3A), TET1/2, TP53) and acquisition of a hypermethylated phenotype, contributing to clonal hematopoiesis of intermediate potential (CHIP) as a significant element for hematological malignancies [4, 137]. The abnormal hypermethylation of the ABL promoter by methyltransferases appears to be a disease hallmark, suggesting genetic instability in CML progenitors [138]. The high level of DNA methylation at promoter site of tumor suppressor gene, *MTSS1*, in LSC leads to its downregulation and an increase in CML LSC proliferation [139]. CML LSCs indicate the increased expression of EZH2, a histone methyltransferase and the catalytic subunit of PRC2, which is associated with reprogramming of tri-methylation of histone H3 (H3K27me3) targets [140]). Aberrant activity of histone deacetylase (HDAC) in CML LSC blocks myeloid differentiation to save LSC [141]. Furthermore, overexpression of Sirtuin 1 deacetylase (SIRT1), part of the HDAC family, regulates the acetylation of numerous transcription factors, including P53, Ku70, and FOXO1, contributing to drug resistance and survival of CML LSCs by increasing the acquisition of genetic mutations [142]. The aberrant expressed protein arginine methyltransferase (PRMT5), which mediates histone methylation for RNA metabolism, can attach to BCR-ABL1 and form a positive feedback loop in the evolution of CML [145]. Indeed, both the inhibition of histone methylation and the modulation of DNA methylation can be a possible target for CML treatment in combination with TKIs.

### MicroRNAs (miRNAs)

There are some evidences of miRNAs concerning their important roles in leukemogenesis and resistance of CML LSCs to treatment with TKI. The quiescent CML LSCs in BM can cause TKI resistance induced by the phosphorylation of SPRED1, increased levels of miR-126, and suppression of a miR-126 modulator [55]. The increased expression of CD70 through blocking miR-29, which activates CD27 and induces WNT signaling [143], as well as the further activation of NFAT leading to increase pro-survival cytokines and IM resistance [144]. When CML LSCs are exposed to IM, their levels of miR-21 rise, leading to TKI resistance [145]. Increases in miR-29a cause downregulation of tumor suppressor TET2 and antioxidant-coding EPAS1, as well as overexpression of anti-apoptotic genes BCL-2 and MCL-1, resulting in greater TKI resistance in CML LSC [146, 147]. In addition, low levels of tumor suppressor miR-142 are related to high levels of oncoproteins such as MCL-1 and c-KIT, which leads to anti-apoptotic, pro-survival, and therapy-resistant effects via reactivating PI3K/AKT, JAK/STAT, and RAS/RAF/MEK/ERK signaling in TKI-resistant LSCs [148, 149]. The expression of miR-30a is reduced following IM therapy, favoring LSC resistance to TKIs via a mechanism that involves Beclin1 and ATG5 [150]. The downregulation of miR‐494‐3p might contribute to the TKI resistance of CML LSCs by reduction in the TKI‐induced apoptosis, therefore be a novel target to effectively eradicate LSCs [147]. The dose-dependent PP2A activation and anti-proliferative functions of miR-300 may be upregulated by BMM signals, which induces growth arrest and expansion of the G0–G1 quiescent CML LSCs. The miR300-induced apoptosis is associated with downregulation of CCND2/CDK6, SET, and other PP2A-regulated CML survival-promoting factors such as JAK2, CTNNB1, Twist1, and MYC; therefore, the inactivation of PP2A is vital for survival of quiescent LSCs. Conversely, BCR-ABL1 downregulates miR300 in CML progenitors to prevent PP2A-mediated apoptosis via the upregulation of *TUG1* long non-coding RNAs (lncRNAs). The modulation of miR300 and/or PP2A-activating treatment can trigger LSC apoptosis; thereby, making this TUG1/miR-300/PP2A signaling pathway important for both CML development and treatment [151]. Therefore, analyzing the mechanisms of miRNA activity in TKI-resistant cases may be beneficial for developing more efficient treatments.

## Therapeutic implications of CML LSCs

In the early 1990s, CML treatment included splenic irradiation and use of cytostatic drugs such as busulfan and hydroxyurea. Later, HSC/BM transplant became the curative treatment, which has certain limitation such as challenge of availability of the compatible donor. In following for long-term survival of BMT cases that showed a continuous relapse over time, interferon alpha (IFN-α) in combination with hydroxyurea was used; however, over time response to them reduced due to resistance and adverse side effects [152]. In recent years, the treatment of CML has significant progression from radiotherapy and conventional chemotherapy to targeted therapies with TKIs and allogeneic hematopoietic stem cell transplantation (allo-HSCT) [153]. Inhibition of BCR-ABL tyrosine kinase activity with TKIs was found to be effective in nearly up to 50% of the vast majority of CML patients in chronic phase (mostly receiving IM), who may stop using TKIs and stay a therapy-free remission. However, patients in accelerated phase or blast crisis are frequently treated with later-generation TKIs and are also candidates for allo-HSCT as a last-resort salvage option [154, 155]. However, the CD34^+^CD38^low^ CML LSCs are less responsive to TKI therapy and can stay quiescent and unaffected for extended periods of time [34], resulting in enhancement of TKI resistance and relapse at the level superior of a molecular response or disease progression [69]. Resistance to any or all TKIs may be provided by ABL1 hotspots mutations such as T315I and T315V, which are mostly susceptible to ponatinib [54]. Meanwhile, TKI application is associated with a number of adverse effects, including myelosuppression, gastrointestinal issues, hepatotoxicity, hyperglycemia, and cardiovascular events [156]. Therefore, utilizing the combination of TKIs and other drugs is of some interest concerning the inhibition of leukemia progression and elimination of CML LSCs.

### Tyrosine kinase inhibitors (TKI) treatments

TKIs competitively bind to the ATP-binding site of the BCR-ABL1 to suppress downstream pathways and leukemogenesis by reducing aberrant phosphorylation of the dysregulated tyrosine kinase [157]. In the 2000s, imatinib mesylate (IM), a first-generation TKI, is quite efficient in apoptosis induction in CML stem cells by lowering programmed death receptor 1 (PD-1) expression on CD8^+^ T cells and monocytic myeloid-derived suppressor cells (MDSCs), resulting in increased cytotoxicity mediated by cytotoxic T lymphocytes (CTLs) and natural killer (NK) cells; however, in another study it showed the enhanced expression of *ATG4B* and survival in CD34^+^ CML cells [158, 159]. Dasatinib, nilotinib (NIL), and bosutinib, as second-generation TKIs (2G-TKIs), are more effective and faster at cytogenetic and molecular response to treatment, but inactive toward T315I mutation. In the T315I mutation, the amino acid at position 315 of exon 6 of the *ABL* gene changed from threonine to isoleucine (Ile), hindering the high affinity binding of the inhibitor to the ATP-binding site of the kinase, thereby producing resistance. The metabolic risk of using second-generation TKI should be considered due to their major side effects [160–162]. Nilotinib is initially based on the IM scaffold and it binds to the same pocket as IM but with a much higher affinity that allows for effectiveness against certain IM-resistant point mutations [18]. Dasatinib, a dual SRC/ABL1 inhibitor, is successful in suppressing STAT5 and reducing mutant TP53, but not in eliminating LSCs [163]**.** Dasatinib increases expression of inhibitory KIR2DL1 receptors that can suppress NK cell toxicity against CML cells [164]. Similar to dasatinib, bosutinib inhibits BCR-ABL1 and SRC family kinases, while is minimally active against KIT and PDGFR [165]**.** Radotinib (RAD) is also a novel potent second-generation TKI with high inhibitory capacity for the BCR-ABL1 oncoprotein [166]. As an alternative 2G-TKIs in patients who are resistant to a 2G-TKI without specific mutations, a third-generation TKI, ponatinib is suggested due to its higher efficacy in eradicating CML stem cells and overcoming TKI resistance by replacing threonine with isoleucine at the ATP-binding site, as well as targeting the VEGFR, KIT, SRC, FGFR, PDGFR, FLT3, and KIT pathways [167]. However, some BCR-ABL1 or compound mutations (for example, T315M, T315V, and Y253H/T315I or E255V/T315I) can create ponatinib resistance [168]. Olverembatinib (HQP1351), a novel orally administered 3G-TKI, was found to be highly effective in CML patients who were resistant to existing TKI treatments, including some with T315I mutations in a phase II trial [169]. Vodobatinib (K0706) is another new orally bioavailable 3G-TKI with promising in vitro action against majority of BCR-ABL mutations, but not T315I. In a phase I trial of patients with CML who had failed to respond to ≥ 3 TKIs or lower, vodobatinib demonstrated an adequate safety profile [170]. Asciminib (ABL001), a FDA-approved fourth-generation TKI, is shown to be efficient against BCR-ABL1-dependent and independent mutations such as T315I with a moderate toxicity potential by blocking binding to the BCR-ABL1 myristoyl pocket (STAMP) and producing therapeutic synergism in lowering CRK-like protein phosphorylation for CML stem cells either as monotherapy or in combination with other TKIs [171, 172]. PF-114, a novel fourth-generation TKI, inhibits BCR-ABL1 and/or STAMP along with suppressing the constitutive activation of PI3K/AKT/ERK1/2 and JAK/STAT3/5 signaling and also elevating p27 levels to inhibit oncogenesis with less toxic complications [173–175]. Rebastinib (DCC-2036) is a newly discovered potent broad-spectrum inhibitor of BCR-ABL1 kinase and many other kinases including SRC kinases and FLT-3, but not KIT. It can inhibit many of the resistant mutants of BCR-ABL, including T315I [176].

### Other drugs, targeting the survival mechanism of CML LSCs

It appears that CML LSCs are not completely reliant on BCR-ABL activity for survival so that these BCR-ABL-independent mechanisms may play a role in disease persistence [11]. Targeting BCR-ABL1-kinase independent pathways could regulate apoptosis, self-renewal, and fate of CML LSCs, also overcome survival pathways (Table 1). Knocking out resistance factors in combination with TKI treatment can sensitize drug-resistant CML stem cells to TKIs and decrease their proportion. Several clinical trials investigate the viability of licensed TKIs when used in combination with certain other agents (Table 2).

**Table 1:** Drugs targeting the BCR-ABL1-independent survival pathways

Drug	Target	Result	Reference
*Targeting autophagy*			
Chloroquine (CQ)	Autophagy	Make CML LSCs susceptible to TKI-mediated apoptosis by blocking lysosome–autophagosome fusion and promoting cellular stress	[244]
Spautin-1	Autophagy	Apoptosis of CML cells via inactivation of the PI3K/AKT pathway and downregulation of anti-apoptotic proteins	[245]
Lys05 (A lysosomotropic drug)	Autophagy gene	Decrease in LSC quiescence and facilitation of myeloid cell growth	[246]
PIK-III	The class III phosphatidylinositol 3-kinase, a vacuolar protein sorting 34 (VPS34)	Reduction of primary CML LSCs numbers	[246]
*Targeting surface antigens*			
IL-1R antagonist	Interaction of interleukin 1 and its receptor	Inhibition of IL-1 signaling (NF-κB) and growth of CML LSC and also sensitize them to nilotinib	[247]
Anti-IL-1RAP antibody	IL-1RAP: Co-receptor of the interleukin 1 receptor (IL1R1)	Killing CML cells and increase in survival of murine xenograft through inhibition of IL-1B signaling and induction of ADCC in CML	[248, 249]
CAR T cell against IL1RAP	IL-1RAP: Co-receptor of the interleukin 1 receptor (IL1R1)	Inhibition of IL-1 signaling (NF-κB) causing antileukemic effects	[250]
vildagliptin	CD26 (DPP4)	Decrease in disease expansion through modulating the dysfunctional SDF1/CXCR4 axis to limit mobilization and niche escape of LSCs	[251]
CAR T cells against CD26	CD26 (DPP4)	Decrease in disease expansion through modulating the dysfunctional SDF1/CXCR4 axis to limit mobilization and niche escape of LSCs	[252]
Drug-conjugated anti-CD33 antibody	CD33	Death of most LSCs in CP-CML resulted from the quick internalization and release of the conjugated drug in the acidic environment of lysosomes and its binding to DNA, resulting in DNA double-strand breaks	[38, 253]
Anti-CD44 antibody	CD44	Inhibition of the adhesion to BMM and dormancy of the LSCs	[254]
IL-3-toxin fusion protein SL-401 (Tagraxofusp)	IL-3R	Antileukemic activity by inhibition of cell growth and induction of cell apoptosis	[255, 256]
CAR T cell against IL-3R (CD123)	IL-3R	Antileukemic activity by inhibition of cell growth and induction of cell apoptosis	[257, 258]
Anti-CD70 antibody	CD70 (CD27L)	Decrease in expression of CD27 and WNT target genes leading to elimination of human CML progenitor/stem cells in combination with imatinib	[143]
*Targeting the interactions with BMM*			
NOX-A12	CXCL12	Prevents LSCs homing and causes TKI sensitization	[259]
Plerixafor (AMD3100)	CXCR4	Prevents LSCs homing and causes TKI sensitization	[260]
Acriflavine	HIF-1	The antileukemic response by the modulation of STAT3/5 signaling Decrease in c-MYC and stemness-related genes (e.g., *NANOG*, *SOX9*, and *OCT4*), and increase in the expression of tumor suppressors (e.g., P57, P19^Arf^, and P16^Ink4a^)	[261] [93, 181]
Thiazolidinediones (TZD) such as Pioglitazone (the anti-type 2 diabetic medication)	PPARγ	Reduction in the STAT5 activity Inhibition of LSC infiltration and localization to the BMM by upregulation of matrix metalloproteinase-9 (MMP-9) and MMP-2 Induction of LSC apoptosis by activation of caspase-3	[182]
Rosiglitazone	PPARγ	Induction of LSC apoptosis associated with the increased expression of the stearoyl-CoA desaturase 1 (SCD1), phosphatase, and tensin homolog (PTEN), and P53	[262]
Clofazimine	Physical interaction with PPARγ to regulate its transcriptional activity	The induction of NF-kB-p65, resulting in P65 destruction, downregulation of peroxiredoxin-1 and increased ROS-induced apoptosis Proteasomal degradation Downregulation of dormancy and self-renewal of CML LSC by suppression of STAT5 expression and consequently downregulation of stem cell maintenance factors (HIF-1α/2α and CBP/P300/CITED2)	[263]
*Targeting signaling pathways*			
Farnesyl transferase inhibitors (FT-Is) such as Tipifarnib and Lonafarnib	Protein farnesyltransferase	Preventing the proper functioning of the Ras	[264, 265]
BP1001 (liposome-incorporated antisense oligodeoxynucleotide)	Growth factor receptor-bound protein 2 (GRB2) (a potent activator of ERK1 and ERK2)	Inhibition of RAS/MEK/ERK pathway	[185]
Trametinib	MEK	Suppression of the MEK/ERK and NF-κB-mediated survival of CML LSCs	[186]
ETC-1907206	MAPK interacting protein 1 and 2 (MNK1/2)	Inhibition of the MAPK interacting protein 1 and 2 (MNK1/2)-eukaryotic initiation factor 4E (eIF4E) pathway and activation of β-catenin	[266]
Dactolisib (NVP-BEZ235)	PI3K and mTOR	Inhibition of CML cell proliferation by triggering autophagy and apoptosis	[267]
Pictilisib (GDC0941)	PI3K	Inhibition of growth and survival of resistant CML cells by induction of apoptosis	[268]
KU-0063794	mTORC1/2	Antiproliferative or proapoptotic effects by inhibiting activation of AKT and other protein kinases	[268]
Rapamycin (sirolimus)	mTOR	Induction of cell growth arrest and apoptosis and a reduction in cell proliferation	[269]
Everolimus (RAD001)	mTOR	Induction of cell growth arrest and apoptosis and a reduction in cell proliferation	[270]
Pimozide	STAT5	A reduction in the proliferation of CML CD34^+^ cells induction of cell cycle arrest and apoptosis	[126]
Ruxolitinib (RUB)	JAK2	Decrease in JAK2/STAT5 activity, reactivation of PP2A	[121]
Fedratinib (TG101348)	JAK2	Decrease in JAK2/STAT5 activity, reactivation of PP2A	[271, 272]
10,058-F4	c-MYC	Promotion of an apoptosis by PP2A reactivation and modulation of autophagy	[273]
OP449	SET	Reactivate PP2A and apoptotic pathways in a PP2A-dependent manner leads to depletion of the LSCs by inhibiting STAT5	[192]
FTY720	SET	Reactivate PP2A and apoptotic pathways in a PP2A-dependent manner leads to depletion of the LSCs by inhibiting STAT5	[191]
BP-5087	STAT3	Overcome independent survival and drug resistance of LSCs	[197]
IFN-α	Activation of STAT1/5	Differentiation and exhaustion of CML stem cells by the upregulation of FAS-R	[196]
PRI-724 (ICG-001)	β-catenin/TCF mediated transcription (WNT/β-catenin signaling)	The disruption of the interaction between CBP and β/γ-catenin, leading to a decrease in self-renewal capability in leukemia-initiating cells in CML	[198, 274]
Misoprostol	PGE1	Activation of this PGE1-EP4 pathway and inhibition of TCF1/LEF1 and FOS/FOSB in WNT signaling	[132]
Niclosamide	Interaction between the FOXM1/β-catenin/NF-Kb	Impairs the ability of CML LSCs to survive and self-renew	[200]
WNT974	PORCN (O-acyl transferase)	Inhibition of WNT signaling suppression of c-MYC, cyclin-D1 and Axin-2 expression, contributing to an increase in the inhibition of proliferation and eradication of CML stem cells	[127, 128]
Cyclopamine	SMO	Eradicating Hh-mediated self-renewal capacity of CML LSC stimulates CML LSCs to cell cycle and become sensitive to TKIs	[202]
LDE225 (sonidegib)	SMO	Eradicating Hh-mediated self-renewal capacity of CML LSC stimulates CML LSCs to cell cycle and become sensitive to TKIs	[203]
Glasdegib (PF-04449913)	SMO	Eradicating Hh-mediated self-renewal capacity of CML LSC stimulates CML LSCs to cell cycle and become sensitive to TKIs	[204]
Vismodegib	SMO	Eradicating Hh-mediated self-renewal capacity of CML LSC stimulates CML LSCs to cell cycle and become sensitive to TKIs	[205]
*Targeting energy metabolism*			
SR-18292	PPARγ coactivator-1α (PGC-1α)	Increase in PGC-1 acetylation, The downregulation of mitochondrial oxidative metabolism Increase in apoptosis of CML CD34^+^CD38^–^ cells	[106]
Tigecycline	mitochondrial metabolism	Impairment of mitochondrial protein synthesis and mitochondrial respiration	[56]
Subutoclax	BCL2	Disruption of energy metabolic pathways and decrease in oxidative phosphorylation levels, resulting in increase in CML LSCs eradication	[208, 209]
*Venetoclax*			
LY25528	BLT2	Inhibition of ALOX15 pathway Inhibition of self-renewal in TKI-resistant CML cells by induction of apoptosis	[211]
Zileuton	ALOX5	Decrease in the survival of CML LSCs in mice	[33]
QLT0267	Integrin-linked kinase (ILK)	Induction of metabolic vulnerabilities by the reduction in the CD36 expression	[212]
*Targeting the epigenetic modification*			
DS-5272	MDM2	The reactivation of P53, silencing ant-iapoptotic MCL-1 and sensitive quiescent CD34^+^ cells to therapy	[215]
Panobinostat (LBH589)	HDAC	Increase in TKI-mediated apoptosis by acetylating HSP90 and increasing proteasomal degradation of key signaling proteins in CML LSCs	[141, 216]
Chidamide	HDAC	Induction of apoptosis by increasing acetylation of histone H3, activation of caspase 3/9, reduction in the β-catenin levels and its downstream targets surviving (a WNT–CBP–β-catenin-regulated gene), and c-MYC	[217, 218]
MAKV-8	HDAC	Reduction in the c-MYC expression and the stimulation of caspase 3/9 and ER stress, all of which contribute to LSC eradication	[219]
Tenovin-6	SIRT1	Increase in apoptosis by increase acetylation of P53	[220, 221]
PJ-68	PRMT5	Induction of CD34^+^CD38^−^ cell apoptosis by inhibiting the WNT/β-catenin pathway and inducing negative control on LSC renewal	[222, 223]

**Table 2:** Complete clinical trials of CML therapies in combination with TKIs

Drug	Target to inhibit	Clinical phase	NCT number	Study population	Year	Purpose
Imatinib + Dasatinib	BCR-ABL oncoprotein	Phase 2	NCT00982488	Patients with CML or Ph ^+^ ALL who treated with dasatinib or imatinib in previous protocols	2009–2016	Evaluate the long-term efficacy and tolerability of dasatinib
Imatinib + Dasatinib	BCR-ABL oncoprotein	Phase 2	NCT00852566	Patients with newly diagnosed CML	2009–2015	Compare the effect of treatment with dasatinib and imatinib on malignant stem cells at 18 months
Imatinib + Dasatinib	BCR-ABL oncoprotein	Phase 3	NCT00481247	Patients with newly diagnosed CP-Ph ^+^ CML	2007–2017	compare the complete cytogenetic response and safety of treatment of dasatinib versus imatinib within 12 months
Imatinib + Bosutinib	BCR-ABL oncoprotein	Phase 3	NCT02130557	Patients with newly diagnosed CML	2014–2020	Investigate the randomized receiving of bosutinib or imatinib and the use of bosutinib as first-line treatment for CML patients
Imatinib + Bosutinib	BCR-ABL oncoprotein	Phase 3	NCT00574873	Patients with newly diagnosed CP-CML	2007–2019	Compare the efficacy and safety of Bosutinib versus imatinib alone
Imatinib + Nilotinib	BCR-ABL oncoprotein	Phase 2	NCT00769327	Patients with early CP-CML	2009–2014	Evaluate the efficacy of nilotinib together with imatinib in treatment
Imatinib + Nilotinib	BCR-ABL oncoprotein	Phase 3	NCT01275196	Patients with newly diagnosed CP-CML	2011–2016	Compare the efficacy and safety of nilotinib versus imatinib alone
Imatinib + Nilotinib	BCR-ABL oncoprotein	Phase 3	NCT02272777	Patients with CP-CML after the end of CAMN107ECN02 study	2014–2019	The extension study followed the CAMN107ECN02 core study (NCT01275196)
Imatinib + Nilotinib	BCR-ABL oncoprotein	Phase 3	NCT00760877	Patients with CP-CML with evidence of persistent Leukemia	2008–2016	Compare the Kinetics of complete molecular response in subjects receiving imatinib or nilotinib therapy
Imatinib + Nilotinib	BCR-ABL oncoprotein	Phase 3	NCT00802841	Patients with CP-CML and suboptimal response to standard dose imatinib	2008–2015	Compare effectiveness of imatinib dose escalation (600 mg once daily) versus nilotinib (400 mg twice daily) in terms of complete cytogenetic response after 6 months
Imatinib + Nilotinib	BCR-ABL oncoprotein	Phase 3	NCT00471497	Patients with newly diagnosed CP-CML	2013–2020	Compare the efficacy and safety of nilotinib versus imatinib
Imatinib + Dasatinib + Nilotinib	BCR-ABL oncoprotein	Phase 2	NCT02709083	Patients with newly diagnosed CP-CML and previously untreated	2016–2018	Assess the efficacy of treatment with first-line dasatinib or nilotinib followed by response guided switch to imatinib
Imatinib + Recombinant Interferon-alpha (INF-α)	BCR-ABL oncoprotein + growth of leukemia cells	Phase 2	NCT00015847	Patients with CML	2003–2011	Investigate the efficacy of combining imatinib with INF-α in the treatment
Imatinib + Interferon-alpha (INF-α) + Cytarabine (ARA-C)	BCR-ABL oncoprotein + growth of leukemia cells	Phase 3	NCT00333840	Patients With Newly Diagnosed Previously Untreated (Ph^+^) CP-CML	2000–2012	Evaluate and compare the side effects and antileukemic benefits of imatinib with those of IFN and ARA-C for patients
Imatinib + Homoharringtonine	BCR-ABL oncoprotein + SMAD3 and TGF-β pathway	Phase 2	NCT00114959	Patients with CML in chronic, accelerated or blast phase who have developed resistance to or have failed previous treatment with Gleevec	2005–2009	Investigate the safety and efficacy of this combination therapy to produce a stronger hematologic or cytogenetic response for a period of 12 cycles in comparison with imatinib alone
Nilotinib + Ruxolitinb	BCR-ABL oncoprotein + alternative pathway independent of BCR-ABL including JAK2/STAT5	Phase 1 and 2	NCT01914484	Patients with Ph ^+^ CML and ALL who have are resistant to prior TKIs	2013–2018	Investigate the effectiveness of a nilotinib and ruxolitinib combination treatment
		Phase 1	NCT01702064	Patients with CP-CML	2013–2019	Determine the maximum tolerated dosage of ruxolitinib as used with nilotinib for therapy
Nilotinib + Sonidegib (LDE225)	BCR-ABL oncoprotein + SMO/Hedgehog signaling pathway	Phase 1	NCT01456676	Patients with CP/AP-CML	2012–2014	Determine the effectiveness of combination nilotinib and LDE225 in treatment
Dasatinib (Sprycel) + Decitabine (Dacogen)	BCR-ABL oncoprotein + Nucleic acid synthesis and expression of certain genes	Phase 1 and 2	NCT01498445	Patients With Accelerated or Blastic Phase CML	2012–2019	Assess whether combining dasatinib and decitabine will potentially affect CML, as well as investigate the optimal dose of Decitabine
Dasatinib (BMS-354825) + Nivolumab (BMS-936558)	BCR-ABL oncoprotein + PD-1 Blocking antibody	Phase1B	NCT02011945	Patients with CML	2013–2020	Dose escalation study in patients with dasatinib and nivolumab to determine safety, tolerability, and preliminary efficacy
Dasatinib + Smoothened (SMO) antagonist (BMS-833923)	BCR-ABL oncoprotein + SMO/Hedgehog signaling pathway	Phase 1 and 2	NCT01218477	Patients with CML	2010–2016	Determine the safety and tolerability result of the combination of BMS-833923 plus dasatinib in CML patients
Dasatinib + Peginterferon-α-2b	BCR-ABL oncoprotein + inducer of immunosurveillance	Phase 2	NCT01872442	Patients With newly diagnosed CP-CML	2013–2018	Investigate the efficacy and safety of dasatinib in combination with low dose of Peg-IFNα-2b as frontline therapy
Imatinib mesylate + panobinostat (LBH589)	BCR-ABL oncoprotein + Histone deacetyation activity	Phase 1	NCT00686218	Patients with previously treated CP-CML	2008–2014	Determine the efficacy and tolerability of Panobinostat combined with imatinib in the treatment of patients
Tyrosine Kinase Inhibitor (TKI) + Pioglitazone (PIO)	BCR-ABL oncoprotein + A diabetic drug against kinase A or kinase B	Phase 2	NCT02730195	CML patients who relapsed Following a First TKI Discontinuation	2016–2019	Assess safety of these drug combination in CML subjects and their survival following a second TKI discontinuation
Tyrosine Kinase Inhibitor (TKI) + Azacitidine (AZA)	BCR-ABL oncoprotein + Changes in genes that are thought to cause leukemia	Phase 1	NCT01460498	Patients With CML Who have Minimal Residual Disease while receiving TKI therapy	2012–2019	Consider the most effective and tolerable dose of Azacitidine that can be administered with a TKI to improve treatment

### Anticancer drugs targeting autophagy

TKI therapy for CML causes autophagy as a drug resistance pathway employed for the survival of LSCs [177]; therefore, suppression of autophagy appears to be a therapeutic option for eliminating LSCs, especially combined with TKI therapy, despite its conflicting consequences in CML. As a result, knocking out either the *ATG5* or *ATG7* genes, which are involved in preautophagosome formation and activation, makes CML LSCs more susceptible to IM [177]. *ATG7* silencing also decreases glucose levels in LSCs, inhibits formation of autophagosome by downregulating signaling pathways (e.g., WNT/catenin, PI3K/AKT/mTORC1, HIF, c-MYC), and activates cytochrome-c/caspase-9/caspase-3, resulting in mitochondrial translation, oxidative phosphorylation (OXPHOS), and oxygen consumption [56]. As mentioned in Table 1, chloroquine (CQ), Spautin-1, Lys05 (a dimeric analogue of chloroquine), and PIK-III, as autophagy inhibitors, can influence the maintenance and function of LSCs**.** Also, inhibition of BMI1, which is a key role in LSC self-renewal and advancement of CML to the acute level through blocking a defensive CCNG2-dependent autophagy pathway, combined with IM significantly reduces the clonogenic properties of CD34^+^ CML cells [178].

### Immunotherapy strategies targeting cell surface markers

Although cell surface markers expressed on CML LSCs and normal HSCs are mostly similar, the expression levels of some markers are much greater in LSCs than in HSCs, providing an opportunity for molecules targeting cell surface receptors of CML LSCs using antibodies. Specific cell surface antigens can be used to develop CML-eradicating immunotherapies such as cell-targeting antibodies, antibody–toxin conjugates, and chimeric antigen receptor-engineered T (CAR-T) cell-based therapy. It seems that they are likely to have less toxic or off-target effects than inhibitors of factors essential for the survival of LSCs. However, unlike AML, CAR cell therapies have not been significantly developed in CML so far [42, 47]. Targeting surface markers particularly overexpressing on CML LSCs such as IL1RAP, CD26, CD44, CD70, and CD123 has demonstrated a powerful antileukemic effect in preclinical CML studies (Table 1), although the viability of some of them in the treatment of patients with CML needs to be further investigated, including anti-CD44 agents with regard to the expression of CD44 on HSCs [179]**.**

### Anticancer drugs targeting the interactions with BMM

Targeting the interaction of SDF1-CXCR4 by NOX-A12 and Plerixafor (AMD3100), CXCR4 antagonist prevents LSC homing and causes TKI sensitization. TKI-resistant mesenchymal cells enhanced BMP4 production, indicating a BMP autocrine loop may cause TKI resistance; hence, new anti-BMP pharmacological molecules could be used to target CML LSCs in their niche [76]. Furthermore, TGF-β stimulates the expression of plasminogen activator inhibitor-1 (PAI-1), a significant physiologic serine protease inhibitor (serpin) of the fibrinolytic network, leading to decrease membrane type-1 metalloprotease (MT1-MMP) activity and mobility of CML LSCs. Therefore, repression of PAI-1 can increase sensitivity of CML LSCs to TKIs through mobilization of CML LSCs from the niche as a measure to combat TKI resistance [180]. Moreover, in hypoxic microenvironment, a HIF-1 inhibitor (acriflavine) along with a TKI may possibly target CML LSCs and be responsible for the antileukemic response [181]. One of the recommended ways of eliminating CML LSCs is the inhibition of PPARγ by pioglitazone, rosiglitazone, and clofazimine. Despite various difficulties, such as adverse effects of PPARγ agonists, the discovery of PPARγ as a novel critical regulator for CML LSC survival offers fresh promises for targeting them [182]. Furthermore, overexpression of PPARα ligands (e.g., clofibrate) and increased expression of human organic cation transporter 1 (hOCT1) by WY-12643 promotes TKI-mediated apoptosis [182, 183]. To draw a conclusion upon these, understanding the interactions between LSCs and the bone marrow niche might lead to a new strategy for the elimination of CML stem cells.

### Anticancer drugs targeting signaling pathways

One of the ways to prevent RAS/MEK/ERK/MAPK pathway is block isoprenoid group transfer as a posttranscriptional modification that induces membrane migration and activation of various proteins, including RAS and RAF by farnesyl transferase inhibitors (FT-Is). Although they show a few benefits, their combination with IM can be useful for CML patients who are not responding to IM monotherapy [184]. The combination of the inhibitors of RAS/MEK/ERK pathway with TKIs can reverse the resistance and cause drug synergistic effect [185, 186]. It appears that reactivating miR-185, which targets PAK6 transcripts, will make therapy-resistant cells more susceptible to TKIs by decreasing MAPK pathway activation, mitochondrial function, ROS production, and autophagy [187].

As evident in Table 1, there are compounds which can also sensitize CML LSCs to TKIs and eliminate them by disrupting the PI3K/AKT/mTOR pathway. Moreover, inhibition of the PI3K/AKT signaling pathway by antagomiR-21 along with IM amplifies programmed cell death 4 (PDCD4) and PTEN, while decreases AKT phosphorylation and MYC expression, which recovers sensitivity of CML LSCs to TKIs by miR-21 depletion [145, 188]. A study showed that combination of IM and upregulation of miR-155 successfully triggered cell death of CD34^+^, CD38^−^ CML stem cells by blocking of PI3K/mTOR pathway [189].

The other potential therapeutic target in combination with TKIs can be the of JAK2/STAT5 pathway and induces cell cycle arrest and apoptosis in CML cells by inhibitors such as pimozide, ruxolitinib, and fedratinib [126]**.** Since JAK2 inactivates PP2A and reactivation of PP2A leads to depletion of the LSCs by inhibiting STAT5, a synergism of JAK2 inhibitors and PP2A activators might be effective in the treatment of CML [190]. Also, MYC inhibitor and a SET antagonist can reactivate PP2A and apoptotic pathways in a PP2A-dependent manner [191, 192]; therefore, the impairment of PP2A inhibitors with a TKI can inhibit STAT5, and deplete LSCs [193, 194]. Combining IM and interferon-γ (IFN-γ) reduces STAT5 phosphorylation, whilst increases STAT1 phosphorylation that increases BCL6 expression and LSC survival [195]. The upregulation of C/EBPb and activation of STAT1 and STAT5 by IFN-α can leads to differentiation and exhaustion of CML stem cells [196]. Moreover, the inhibition of STAT3 by OP449 has a strong potential to stops TKI-resistant CML LSCs survival [197].

The disruption of the interaction between CBP and β/γ-catenin by PRI-724 can cause a decrease in self-renewal capability in leukemia-initiating cells in CML and eradication of drug-resistant primary CML cells [198]. In addition, CBP inhibitor therapy increases β-catenin binding to P300, which mediates cell differentiation, and causes P53/P21-dependent senescence in BCR-ABL mutant CML cells [131]. Inhibition of PGE2 by blocking the activity of cyclooxygenase-2 leads to decrease in both β-catenin and resistance to TKIs [199]. However, PGE1 shows protective roles against LSCs by suppression AP-1 factors such as FOSB that make it a target for CML stem cell eradication [33, 132]. Modulation of WNT signaling and other signaling pathways such as mTORC1, STAT3, NF-kB, and Notch pathways, by niclosamide impairs the ability of CML LSCs to survive and self-renew by disrupting [200]. Furthermore, the increased level of tyrosine phosphatase FAS-associated phosphatase 1 (FAP-1), which targets a β-catenin inhibitor (GSK3β), mediates persistence of CML LSCs [201]. The combination of porcupine (PORCN) inhibitor (WNT974) with NIL in transgenic mice models showed the inhibition of WNT/β-catenin signaling and suppression of c-MYC, cyclin-D1, and Axin-2 expression, contributing to an increase in the inhibition of proliferation and eradication of CML stem cells [127, 128].

Targeting the Hh signaling pathway by using the SMO inhibitors, (e.g., cyclopamine and LDE225 (sonidegib)), alone or in combination with TKIs, can be an effective treatment strategy for eradicating Hh-mediated self-renewal capacity of CML LSC and suppress their growth [202, 203]. The overexpression of GLI2 enhances the generation and commensurate of dormant LSCs, and also increases the repression of cell cycle regulatory genes, therefore providing glasdegib (PF-04449913), and an antagonist of the GLI2 transcriptional activator (SMO) stimulates CML LSCs to cell cycle and become sensitive to TKIs [204]. The inhibition of Hh pathway by vismodegib can treat the upregulation of autophagy in CML cells; hence, the combination of vismodegib with silencing of *ATG5* and *ATG7* can cause significant increase in CML cell death [205]. Moreover, overexpression of miR-326 results in an increased rate of apoptosis in CML CD34^+^ cells by downregulation of SMO [206].

### Anticancer drugs targeting energy metabolism

The inhibition of mitochondrial function has antileukemia effects along with TKIs [90]. The important findings suggest that SIRT1 and/or elements downstream in the OXPHOS pathway may be potential therapeutic targets, for instance, inhibiting PPARγ coactivator-1α (PGC-1α) by SR-18292 in combination with TKIs could avoid CML relapse by the increase in on PGC-1 acetylation levels affected of SIRT1 [106].

As a result of shifting to mitochondrial metabolism, TKI-tolerant cells showed vulnerability to mitochondrial-druggable targets with antileukemia effects such as inhibition of the first enzyme in glutamine-dependent mitochondrial metabolism combined with TKI [207]. CML LSCs have also represent susceptibility to TKIs and tigecycline, an antibiotic from glycylcycline class, impairing mitochondrial protein synthesis and the mitochondrial respiration [56]. Furthermore, BCL-2 inhibitors (subutoclax and venetoclax) can increase the eradication of CML LSCs by disrupting energy metabolic pathways [208, 209]. Recently, an innovative strategy based on a liposome loaded with the BCL2 inhibitor (venetoclax) using an anti-CD26 antibody (begelomab) to selectively target CML LSCs showed that the immunoliposome could reduce cell growth and also induce apoptosis in CD26^+^ LSCs, along with synergistic effects by coadministration with imatinib or nilotinib [210]. Interestingly, inhibition of ALOX15 pathway involved in fatty acid metabolism with the BLT2-specific inhibitor triggered apoptosis and inhibited self-renewal in TKI-resistant CML cells in blast crisis phase [211]. Although blocking ALOX5 with zileuton decreases the survival of CML LSCs in mice, targeting ALOX5 is unlikely to be successful due to the low expression of ALOX5 in humans [33]. In another study, it was indicated that targeting integrin-linked kinase (ILK), which is highly upregulated in CML LSCs, can induce metabolic vulnerabilities by reduction in the CD36 expression, as a fatty acid receptor; therefore, an ILK inhibitor combined with dasatinib can increase the chance of recipient's survival [212].

### Anticancer drugs targeting the epigenetic modification

Inhibition of EZH2 and its downregulation by TKI therapy blocks survival of leukemia-initiating cells and promotes CML LSCs apoptosis without impairing normal HSCs by modulation PTEN expression. Hence, inhibiting EZH2 along with TKI therapy increases activation of H3K27me3 targets such as CDKN2A and upregulates pro-apoptotic targets of P53 (e.g., NOXA, P53 upregulated modulator of apoptosis (PUMA), BAX, CDKN2A, TNFRS10B), causing TKI sensitivity to be restored and CML LSCs to be eradicated [70, 213]. The reactivation of P53 by an HDM2 antagonist or an MDM2 inhibitor restores sensitivity of quiescent CD34^+^ cells to BCL2 inhibitor and TKI-induced apoptosis [99, 214, 215]. Moreover, MI-219 can induce apoptosis through the alteration of HDM2 function, reactivation of P53 mediated by downregulation of c-MYC and upregulation of P21, and diminished essential genes for LSC self-renewal [99, 214, 215].

HDAC inhibitors, panobinostat, combined with TKIs can disrupt LSC quiescence and increase TKI-mediated apoptosis by acetylating HSP90 and increasing proteasomal degradation of key signaling proteins in CML LSCs [141, 216]. Another HDAC inhibitor, chidamide, induces apoptosis by increasing acetylation of histone H3, activation of caspase 3/9, reduction in the β-catenin levels by inhibiting the WNT–CBP–β-catenin pathway with limited toxicity to normal HSCs [217, 218]. Novel pan-HDAC inhibitor, MAKV-8, also contributes to LSC eradication through the stimulation of caspase 3/9 and ER stress [219]. Targeting SIRT1 in CML LSCs by Tenovin-6 or TV39OH leads to enhance apoptosis by increase acetylation of P53 [220, 221]. A PRMT5 inhibitor can induce apoptosis of CD34^+^CD38^−^ cells by inhibiting the WNT/β-catenin pathway and inducing negative control on LSC renewal [222, 223].

### Gene therapy

Advances in molecular biology and genetics have expanded our knowledge of genes involved in disease development. In line with this, recently, the common techniques used to monitor CML patients, detect the Ph chromosome, and recognize the *BCR-ABL1* transcript are conventional cytogenetics and fluorescence in situ hybridization (FISH), and reverse transcriptase-polymerase chain reaction (RT-PCR) [60, 224, 225]. RT-PCR is also used to assess the following molecular response to treatment, defined as the ratio of *BCR-ABL1* to *ABL1* transcripts (known as molecular response (MR)) and categories in different groups including complete cytogenetic remission (MR ≤ 1%), major molecular response (MMR) (MR ≤ 0.1%), deep molecular response (DMR) ( MR4 ≤ 0.01%), and molecularly undetectable leukemia MR4.5 ≤ 0.0032% [60, 164].

Advances in genomic techniques have also led to the development of highly translational murine models of human hematologic malignancies [226]. In fact, the ideal murine model should replicate the genetic and molecular heterogeneity of tumors in immune-competent mice, while also offering a mechanism of monitoring clinical behavior of the human disease, progression, and treatment efficacy [227]. Xenograft models are highly beneficial for determining the efficacy of therapeutics on human tumor cells by proofing the concept by in vitro studies within in vivo conditions. However, there are some limitations that should be considered such as the lack of tumor microenvironment, inability to determine tumor interaction with the immune system, and inability to test complex genomic interactions in a single-cell system [226]. The transgenic mouse model has been used to mimic human cancers with an etiology based on genetic aberration by injecting a gene of interest in the vector form into a fertilized egg, allowing investigation of both microenvironment and immunity on the response of tumor in either preventative or long-term therapy. However, the manipulation of embryonic stem cell could associate with the potential of off-target mutation and genetic alteration during development. They are also genetically not as complex as human tumors [228, 229]. For the first time, the induction of CML-like myeloproliferative syndrome was observed in irradiated recipient mice transplanted with a retroviral vector encoding the BCR-ABL1 fusion protein to identify regions in this oncoprotein that are important for CML transformation in mice and design of TKIs [230]. Mouse models of retroviruses may evaluate the function of individual genes for CML development and progression, including the expression of STAT5 that is necessary for BCR-ABL1-mediated leukemogenesis [231]. Although the CP-CML depends on BCR-ABL1, the progression of acute blast crisis is mediated by additional genetic changes and mouse models of CML are needed to develop therapies for unresponsive patients to TKIs [232]. At present, CML could be induced in most of the inbred mouse strain, including C57BL/6, BALB/c, and viable gene knockout mice strain with high efficiency, providing an excellent model for studying CML LSCs and evaluating therapeutic agents for CML treatment based on their short latency (about 3 weeks) [233]. Therefore, retroviral mouse models have not only been instrumental in identifying the mechanisms of leukemogenesis but have also contributed to advances in the understanding of disease progression in CML and the identification of new therapeutic targets.

In general, TKIs only inactivate the oncoprotein, but the oncogene continues unaffected and treatment discontinuation is only an option for a small subset of patients; therefore, the interruption/deletion of the oncogenic sequence might be an effective new therapeutic option [224]. The emergence of clustered regularly interspaced short palindromic repeats (CRISPR)-Cas9 technology can be a treatment based on its capacity to induce a specific DNA double-strand break in precise locations and providing complete and permanent oncogene knockout by generating an animal carrying highly targeted genetic modification [224, 234]. Additionally, this system can be used to deliver combinations of guide RNAs to modify multiple genes in a single mouse hematopoietic stem cell, to more closely model the complexity of hematopoietic malignancy [235]. Although the risk of off-target editing is seen in cell-based systems, the accuracy of the CRISPR-mediated editing system is suggested in the embryonic system [236]. In 2015, the first CRISPR/Cas9 system that could correct acquired mutations in a human myeloid leukemia cell line was demonstrated and then successfully used in animal [237]. Three years later, the first clinical trial focused on HSCs by using CRISPR-Cas9-modified HSCs was approved [238]. Furthermore, there were mouse models for human HSC engrafting and mimicking human CML to provide opportunities to evaluate these CRISPR/Cas9 therapeutic applications [239]. In 2017, the CRISPR/Cas9 system effectively abrogates the *BCR/ABL1* oncogene and reversed the tumorigenicity in edited CRISPR cells in a CML xenograft animal model [240]. In the following, other genome-editing nucleases (e.g., zinc finger nucleases (ZFN) targeting the exon 1 of BCR) achieved the abrogation of the BCR/ABL1 oncogene in both sensitive and resistant forms of K562 to IM [241]. Recently, for the first time, a CRISPR/Cas9 short deletion system interrupts the *BCR/ABL1* oncogene in primary leukemic stem cells Sca1^+^ from a CML mouse model and CD34^+^ from human CML patients. They showed that edited LSCs had impaired tumorigenic activity and capacity for multipotency and also confer a significant therapeutic benefit on CML mouse models [242]. In addition to BCR/ABL1 oncogene, other genes involved in CML LSCs survival such as EZH2 can make LSCs more susceptible to TKIs by using CRISPR/Cas9-mediated gene editing [140]. Although the CRISPR-Cas9 system induces double-strand breaks at target sites in genomic DNA, it can also generate undesirable cleavages at off-target sites leading to mutations and the disruption of normal genes [243].

## Conclusion

Over the past years, the inadequacy of many CML therapies results from their failure to target LSCs represents CML LSCs as the most critical target in the treatment. However, they are also the most difficult population to be targeted due to both their heterogeneity and phenotypic similarities with HSCs [9]. CML LSCs can be resistant to TKI therapy and remain after therapy, serving as a reservoir for residual disease and relapse due to independent BCR-ABL activity including the various molecular mechanisms and signaling pathways, which are often activated by epigenetic mechanisms and the impact of the bone marrow niche, as mentioned earlier in this study. Further investigating these different aspects of CML LSCs maintenance might enable us to provide novel therapeutic targets for the development of a TKI-based combinatorial therapy to eradicate CML stem cells. Altogether, a promising therapeutic strategy would combine TKI with drugs targeting alternative survival pathways. However, some of scientific research provides questionable or contradictory values, remained to be clarified to draw a proper conclusion.

## Data Availability

Not applicable.

## Abbreviations

ABL: Abelson murine leukemia virus
AP: Accelerated phase
ALL: Acute lymphoblastic leukemia
ADAR1: Adenosine deaminases acting on double-strand RNA1
AMPK: AMP-activated kinase
ALOX5: Arachidonate lipoxygenase
BCL-XL: B-cell lymphoma-extralarge
BCP: Blast crisis phase
BMM: Bone marrow microenvironment
BMPs: Bone morphogenic proteins
BCAA: Branched-chain amino acids
BCR: Breakpoint cluster region
CAR-T: Chimeric antigen receptor-engineered T
CML: Chronic myeloid leukemia
CP: Chronic phase
JNK: c-JUN N-terminal kinase
CBP: CREB-binding protein
CDK1: Cyclin-dependent kinase 1
DMR: Deep molecular response
DLAT: Dihydrolipoamide S-acetyltransferase
DPP4: Dipeptidyl peptidase-4
DYRK2: Dual specificity tyrosine phosphorylation regulated kinase 2
EGFR: Epidermal growth factor
ERK: Extracellular signal regulated kinase
FAP-1: FAS-associated phosphatase 1
FGF2: Fibroblast growth factor 2
FISH: Fluorescence in situ hybridization
FLT3: FMS-like tyrosine kinase 3
FOXO: Forkhead box O
FZD4: Frizzled-4
GLUT1: Glucose transporter 1
GSK3: Glycogen synthase kinase 3 beta
G-CSF: Granulocyte colony-stimulating factor
GRB2: Growth factor receptor-bound protein 2
Hh: Hedgehog
HSC: Hematopoietic stem cell
HGF: Hepatocyte growth factor
HHT: Homoharringtonine
hOCT1: Human organic cation transporter 1
HIFs: Hypoxia-inducible factors
IKZF1: IKAROS family zinc finger 1
IM: Imatinib mesylate
ILK: Integrin-linked kinase
ICAM-1: Intercellular adhesion molecule-1
IFN-α: Interferon alpha
IFN-γ: Interferon-γ
IL1R1: Interleukin 1 receptor-1
JAK: Janus kinase
KLF4: Krüppel-like factor 4
LSCs: Leukemia stem cells
LTB4: Leukotriene B4
LKB1: Liver kinase B1
lncRNAs: Long non-coding RNAs
LTβR: Lymphotoxin-β receptor
MMR: Major molecular response
MMP-9: Matrix metalloproteinase-9
MSCs: Mesenchymal stromal/stem cells
MVs: Microvesicles
miRNAs: MicroRNAs
MAPK: Mitogen-activated protein kinase
Msi2: Musashi 2
MDSCs: Myeloid-derived suppressor cells
NK cells: Natural killer cells
NIL: Nilotinib
NFAT: Nuclear factor of activated T-cells
CRISPR: Clustered regularly interspaced short palindromic repeats
OMA: Omacetaxine mepesuccinate
OXPHOS: Oxidative phosphorylation
PUMA: P53 upregulated modulator of apoptosis
PPARγ: Peroxisome proliferator-activated γ
Ph: Philadelphia (Ph) chromosome
PTEN: Phosphatase and tensin homolog
PI3K: Phosphoinositide 3-kinase
PI3K: Phosphatides inositol 3 kinase
PAI-1: Plasminogen activator inhibitor-1
PGC-1α: PPARγ coactivator-1α
PD-1: Programmed death receptor 1
PML: Promyelocytic leukemia
PGE2: Prostaglandin E2
PKC: Protein kinase C
PP2A: Protein phosphatase 2A
PDH: Pyruvate dehydrogenase
RAD: Radotinib
ROS: Reactive oxygen species
RT-PCR: Reverse transcriptase-polymerase chain reaction
RUB: Ruxolitinib
SR-B2: Scavenger receptor-B2
SHC: SRC homology 2 domain-containing
SCFR: Stem cell factor receptor
TZD: Thiazolidinediones
TGF-β: Transforming growth factor-β
PKM2: Tumor M2-pyruvate kinase
TKI: Tyrosine kinase inhibitor
VPS34: Vacuolar protein sorting 34
VCAM-1: Vascular cell adhesion molecule-1
WHO: World Health Organization
ZFN: Zinc finger nucleases
